# The Hidden Hand of Asymptomatic Infection Hinders Control of Neglected Tropical Diseases: A Modeling Analysis

**DOI:** 10.1093/cid/ciae096

**Published:** 2024-04-25

**Authors:** Kat S Rock, Lloyd A C Chapman, Andrew P Dobson, Emily R Adams, T Déirdre Hollingsworth

**Affiliations:** Zeeman Institute for Systems Biology and Infectious Disease Epidemiology Research (SBIDER), University of Warwick, Coventry, United Kingdom; Mathematics Institute, University of Warwick, Coventry, United Kingdom; Department of Mathematics and Statistics, Lancaster University, Lancaster, United Kingdom; Centre for Mathematical Modelling of Infectious Diseases, London School of Hygiene and Tropical Medicine, London, United Kingdom; Department of Ecology and Evolutionary Biology, Princeton University, Princeton, New Jersey, USA; Santa Fe Institute, Santa Fe, New Mexico, USA; Department of Clinical Sciences, Liverpool School of Tropical Medicine, Liverpool, United Kingdom; Nuffield Department of Medicine, Big Data Institute, Li Ka Shing Centre for Health Information and Discovery, University of Oxford, Oxford, United Kingdom

**Keywords:** asymptomatic infection, Chagas disease, *gambiense* sleeping sickness, visceral leishmaniasis, modeling

## Abstract

**Background:**

Neglected tropical diseases are responsible for considerable morbidity and mortality in low-income populations. International efforts have reduced their global burden, but transmission is persistent and case-finding-based interventions rarely target asymptomatic individuals.

**Methods:**

We develop a generic mathematical modeling framework for analyzing the dynamics of visceral leishmaniasis in the Indian sub-continent (VL), *gambiense* sleeping sickness (gHAT), and Chagas disease and use it to assess the possible contribution of asymptomatics who later develop disease (pre-symptomatics) and those who do not (non-symptomatics) to the maintenance of infection. Plausible interventions, including active screening, vector control, and reduced time to detection, are simulated for the three diseases.

**Results:**

We found that the high asymptomatic contribution to transmission for Chagas and gHAT and the apparently high basic reproductive number of VL may undermine long-term control. However, the ability to treat some asymptomatics for Chagas and gHAT should make them more controllable, albeit over relatively long time periods due to the slow dynamics of these diseases. For VL, the toxicity of available therapeutics means the asymptomatic population cannot currently be treated, but combining treatment of symptomatics and vector control could yield a quick reduction in transmission.

**Conclusions:**

Despite the uncertainty in natural history, it appears there is already a relatively good toolbox of interventions to eliminate gHAT, and it is likely that Chagas will need improvements to diagnostics and their use to better target pre-symptomatics. The situation for VL is less clear, and model predictions could be improved by additional empirical data. However, interventions may have to improve to successfully eliminate this disease.

Neglected tropical diseases (NTDs) have a major impact on human health and economic development [1]. A substantial component of this is the high proportion of asymptomatically infected individuals who may transmit infection while not developing, or only slowly developing, symptoms that qualify them for treatment [2]. A cruel irony is that treatment can be more effective when patients are asymptomatically infected, as has been shown for Chagas disease in the Americas [3–5]. Here we focus on 3 major vector-borne NTDs on different continents: visceral leishmaniasis (VL) in the Indian subcontinent, *gambiense* human African trypanosomiasis (sleeping sickness [gHAT]), and Chagas disease in the Americas. We use a parameterized mathematical model to estimate the relative contribution of asymptomatic individuals to onward transmission, based on our current understanding of the transmission dynamics of these diseases. As NTDs, there are, of course, limitations to our understanding of these transmission patterns. Although intervention programs exist for each disease, they typically do not address asymptomatic contributions to infection and this may appreciably undermine control gains [6–9]. We develop a general model that quantifies the impact of the transmission by asymptomatic hosts on the long-term sustainability of intervention programs. The analysis illustrates the importance of tackling asymptomatic infection using better diagnostic tools, new treatment options, and vector control.

Many pathogens have long asymptomatic periods when transmission may occur, and the host is unaware that they are infected. Coronavirus disease 2019 (COVID-19) and human immunodeficiency virus (HIV) are notable examples [10, 11], but several important vector-borne diseases also have these properties, notably VL, gHAT, and Chagas disease [2]. These hidden reservoirs of infection present a challenge to public health, particularly if asymptomatic individuals are major contributors to transmission. Recognizing and quantifying the magnitude of this problem is likely to lead to notable modification of current public health policies, demonstrating a need to: (i) more systematically diagnose and treat asymptomatic hosts and (ii) reduce transmission from asymptomatic hosts using suitable interventions.

Our goal is to address 3 questions: (a) How prevalent are asymptomatic infections in VL, gHAT, and Chagas disease and how infectious are they to vectors? (b) What is their relative contribution to transmission compared to hosts with overt symptoms? (c) How does a better understanding of the role of asymptomatic infections inform attempts to control and eliminate vector-borne NTDs and other similar, or emerging, pathogens?

## EPIDEMIOLOGY AND CONTROL

**Figure 1.**
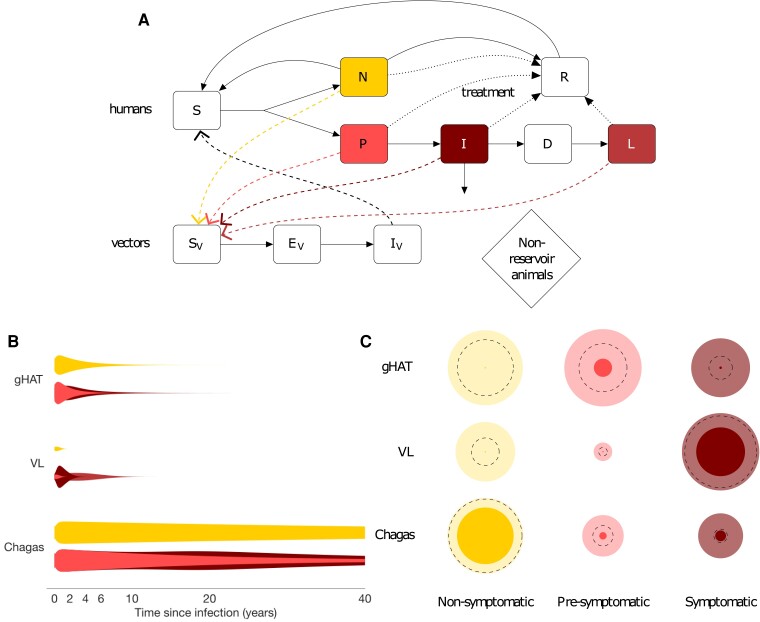
*A*, Flow diagram for generic model of transmission of vector-borne kinetoplastid disease showing progression of susceptible individuals (*S*) to asymptomatic infection following infection by infectious vectors (*I*_V_) and infection of susceptible vectors (*S*_V_) by infectious individuals. Asymptomatics are divided into two groups who are usually indistinguishable by standard diagnostics, non-symptomatics (*N*) and pre-symptomatics (*P*) who go on to develop symptoms (*I*). For visceral leishmaniasis (VL) only, some treated infections progress to a dormant phase (*D*), followed by post kala-azar dermal leishmaniasis (PKDL; *L*). Disease progression is shown by solid arrows, transmission by dashed arrows, and treatment by dotted arrows. *B*, Schematic of relative infectiousness (represented by the width of violin plots) of non-symptomatic, pre-symptomatic and symptomatic infection over time since infection for *gambiense* human African trypanosomiasis (gHAT), VL in the Indian sub-continent and Chagas disease. Schematics derived from a handful of parasitological studies and limited xenodiagnostic studies (which assess transmission from infected individuals toward vectors) and epidemiological studies of the natural history of the disease, alongside indications that symptoms are generally associated with higher parasite loads (Supplementary Data 1). Uncertainty in durations is shown by thin tails. *C*, Estimated relative contribution (represented by bubble sizes) to *R*_0_^2^ (secondary infections) of non-symptomatic, pre-symptomatic and symptomatic infection for the three diseases in the absence of interventions. PDKL is assumed to only occur in the presence of treatment so is not represented in this plot. Darker inner and lighter outer bubbles represent the minimum and maximum possible contribution to *R*_0_^2^ based on lower and upper bounds for parameters from the literature. Dotted lines represent contributions based on mid-range, illustrative values of parameters from the literature (see Supplementary Data 1 and Supplementary Text 1).

VL, gHAT, and Chagas disease are vector-borne parasitic diseases caused by flagellated protozoa belonging to the Class Kinetoplastida. They are the subject of broad-scale interventions (see Supplementary Table 1) with targets for elimination as a public health problem (Chagas and VL) and elimination of transmission (gHAT) by the end of 2030 [12]. All 3 are considered fatal if left untreated in the symptomatic form, and all 3 have substantial and (largely) untreated asymptomatic populations with uncertain natural history [3, 13–15]—this has impeded previous analyses of their impact on transmission. One great challenge is that the definition of asymptomatic infection varies according to the diagnostic used and therefore changes over time [16, 17]. Here we have defined asymptomatic infection as either pre-symptomatic, for those who go on to develop disease, or non-symptomatic, for those who never develop symptoms (see disease-specific definitions in Supplementary Table 2). We have collated the relatively limited available parasitological, epidemiological and xenodiagnostic data to quantify their natural history of disease (Supplementary Data 1, Figure 1*B* ), which we combine with estimates of the proportion of infections in each group to estimate the potential contributions of each group to transmission (Figure 1*C* ).

### Epidemiological Metrics

Three key metrics are used throughout the present study to evaluate the potential impact of different interventions on each disease and compare and contrast the different diseases:

- the full-cycle (host-to-host) basic reproduction number, *R*_0_^2^, for a vector-borne disease, defined as the number of secondary infections generated by a single infectious individual in an entirely susceptible population, and the full-cycle control reproduction number, *R*_c_^2^, the same quantity accounting for control interventions;
- the contribution of asymptomatics to *R*_0_^2^, denoted θ, and that specifically from pre-symptomatics θ_P_ and non-symptomatics θ_N_;
- the contribution of asymptomatics, and that of pre-symptomatics and non-symptomatics, to transmission over time.

### gHAT

*Trypanosoma brucei gambiense* is transmitted by the bites of infected tsetse in sub-Saharan Africa. Due to robust intervention programs, prevalence has recently been brought to low levels, and fewer than 800 annual global cases were reported during 2020–2022 [18]. For gHAT most symptomatic infections present within a few years (Figure 1*B* ), although there are much longer examples [19]. There is evidence of trypanosomes circulating in skin and other organs [20], and one study shows that animals/mice with no detectable blood parasites can still infect tsetse through their skin at ∼58% of the frequency compared to those with blood infections [21]. The current drug options preclude treatment of serological suspects without visualization of the parasite (even if symptomatic), but symptoms are not required to administer treatment. To date, analyses of gHAT transmission have not been able to quantify the extent to which asymptomatics hinder efforts to achieve elimination, although modeling increasingly suggests that systematically undetected human infections are driving transmission [17, 22–24].

In a fully susceptible population, the relative contribution of each type of infection to transmission is essentially the product of the proportion of infections it represents, the average duration of infection and its relative infectiousness to the vector. When calculating this quantity for gHAT, available data suggest that pre- and non-symptomatic individuals are contributing to a large proportion of transmissions (Figure 1*C* , Supplementary Data 1). Current screening and vector control activities reduce transmission by targeting all 3 groups, but imperfect diagnostic algorithm sensitivity (especially for skin infections [22]) and non-participation of individuals who do not feel sick in active screening [25] could hinder the efficiency of the interventions targeted at humans. Vector control activities, primarily using Tiny Targets, are very effective at reducing tsetse populations [26] but are not conducted in all endemic settings.

### VL

*Leishmania donovani* is transmitted by female sandflies. Incidence of the disease in the Indian sub-continent (ISC), where there is an active elimination campaign, has decreased dramatically (falling from 37 000 cases in 2011 to 1500 cases in 2021 [18]). Infection is characterised by a long pre-symptomatic period, and many individuals who are asymptomatically infected (Figure 1*A* ). Recent xenodiagnostic studies [27, 28] suggest that asymptomatically infected individuals are significantly less infectious to sandflies than symptomatic VL cases (with a probability of transmission of <2% compared to 67%–78% for VL cases). Nevertheless, experimental data suggest that these studies may have limited sensitivity to detect transmission to sandflies [29], and outbreaks of VL in regions where there are asymptomatic individuals but virtually no VL cases have been reported, suggesting asymptomatic individuals can transmit to sandflies [30, 31]. Post kala-azar dermal leishmaniasis (PKDL), a skin infection of *Leishmania* parasites, which mostly occurs after treatment for VL, is an added challenge in controlling transmission of VL because PKDL cases constitute a potentially large and untreated reservoir of infection, especially as the incidence of VL decreases [32], as some cases are infectious to sandflies [27, 33]. Here we explicitly include PKDL in our model, to assess the role that it plays in VL transmission as VL incidence decreases under interventions. Previous modeling analyses have suggested that asymptomatic individuals are likely to be far less infectious to sandflies than symptomatic cases (1/80th–1/40th as infectious) but may be a major contributor to overall disease transmission because they represent a large proportion of the infected population [34–36] particularly once immunity declines in the population post-outbreaks [37, 38]. Without addressing these putative infection reservoirs, the gains made during the elimination campaign may be compromised.

In contrast to gHAT, the currently available estimates suggest that symptomatic VL infection is the largest contributor to transmission, stressing the importance of effective case finding (Supplementary Data 1, Figure 1*C* ). However, there is large uncertainty in the proportion of infections that progress to disease and in the relative infectiousness of different infection states. The main interventions against VL currently employed in the Indian subcontinent are early detection and treatment of symptomatic cases and indoor residual spraying of insecticide (IRS) aimed at reducing vector abundance [39, 40]. There is currently no treatment available for asymptomatic infection due to the high toxicity and potential side effects associated with VL and PKDL treatment drugs [41].

### Chagas Disease

Chagas disease is endemic in South America and spreading to the United States, Europe, and Southeast Asia following migration of asymptomatically infected human hosts. It is associated with poor housing because the triatomine bugs that transmit the disease live in cracks in the walls of rural mud houses. Bed bugs may act as vectors in areas where triatomines are controlled or absent [42]. There is a large reservoir of infection in wildlife, and multiple species of triatomines transmit the pathogen [43], but much of the incidence is believed to be driven by a very large pool of asymptomatic human infection in areas of anthroponotic transmission [44]. The triatomine vectors are particularly efficient at acquiring infection from asymptomatic hosts, whence their widespread use in xenodiagnoses [45]. Differentiation of asymptomatic infection and disease is essential since the period before symptom onset is typically several decades (Figure 1*B* ); the symptomatic stage is potentially fatal, even after several years of treatment. An additional challenge related to the asymptomatic nature of Chagas disease is the contamination of blood banks in endemic and non-endemic countries when donors do not know their infection status. This problem is becoming particularly important in areas where asymptomatic migrants from South America join the labour force in the United States, Europe, Australia, and Southeast Asia [46]. Modeling has shown that identification and treatment of asymptomatics can substantially reduce the prevalence of Chagas and aid elimination prospects [7]. The relative contributions to transmission, derived from available literature (Supplementary Data 1, Figure 1*C* ), clearly support the view that asymptomatic infection is a major contributor to transmission, even if it is only 33% as infectious as symptomatic infection, due to its long duration. Historically, vector control through IRS has been the main strategy used to reduce transmission of Chagas, but increasing availability of anti-trypanosomal drugs has enabled broader treatment, including for chronic infection in adults [3, 7].

For all 3 diseases, there is uncertainty in the relative contributions of non-symptomatics, pre-symptomatics and symptomatics due to the wide ranges of the parameter values in the literature (Supplementary Data 1). This emphasises the need for better empirical data and diagnostics, because the contributions of the different states could be roughly similar, or differ by orders of magnitude.

Although there are marked differences in the epidemiology of these diseases, particularly the timescale for disease progression, they share important features in terms of the public health relevance of the role of asymptomatics and the need for quantitative investigation of their dynamics. The paucity of experimental data increases our dependence upon mathematical modeling to predict the impact of the asymptomatic population on interventions as we move towards the peri-elimination era. We group the characteristics of asymptomatic infection across the diseases to see if generalisable themes that are directly relevant to intervention measures arise from a cross-disease comparison using a generic transmission model (see Supplementary Text 1).

## METHODS

**Figure 2.**
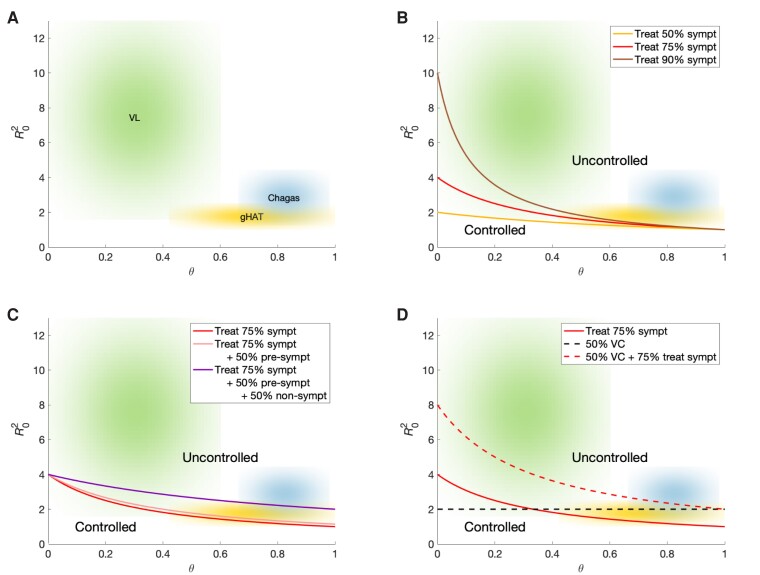
Potential for control of VL, gHAT, and Chagas disease under different combinations of generic interventions. *A*, Plot illustrating the relative position of each of VL (green), gHAT (yellow), and Chagas disease (blue) in the phase space determined by the full-cycle reproduction number of the disease, *R*_0_^2^ (the average number of secondary infections caused by one infectious individual in an entirely susceptible population), and the proportional contribution of non-symptomatic and pre-symptomatic individuals to *R*_0_^2^, *θ*. Clouds represent parameter uncertainty. *B*–*D*, Sensitivity of the diseases to different interventions. The curves delineate successful control (below the line) with each intervention: (*B*) treating symptomatic individuals; (*C*) treating symptomatic individuals and pre-symptomatic and/or non-symptomatic individuals; (*D*) vector control (VC)—achieving 50% vector control corresponding to halving the vector population, reducing the bite rate or shortening vector life expectancy. Abbreviations: gHAT, *gambiense* human African trypanosomiasis; VL, visceral leishmaniasis.

We build upon insights developed by Fraser et al [47] and consider the role of interventions that differentially impact the asymptomatic pool: we first use a flexible, generic deterministic, compartmental model that explicitly includes the natural history of infection, with compartments for non-symptomatics and pre-symptomatics, and also transmission via vectors (Figure 1*A* ). Using this framework we consider the relationship between the basic reproduction number for a vector-borne disease, *R*_0_^2^, and *θ*, the proportion of new infections arising from both non-symptomatic and pre-symptomatic individuals (Figure 2). Reproduction numbers below one will eventually lead to elimination of infection, whereas values above this threshold mean new infections will continue. We performed our simulations using MATLAB 2021b (see Supplementary Code 1 and 2) to compute plausible *θ* ranges for the different diseases given information collated from the literature (see Supplementary Data 1), and this software was also used to numerically solve our ODE model system (described in Supplementary Text 1) under a range of interventions for each disease.

## RESULTS

Broadly speaking, in the absence of effective interventions to combat non-symptomatic and pre-symptomatic infections (diseases in the upper right-hand corner of the plots in Figure 2 with higher *R*_0_^2^ and higher *θ*) are harder to control. Even in the presence of uncertainty in the natural history, our estimates suggest treatment solely of symptomatic individuals for gHAT is unlikely to interrupt transmission (Figure 2*B* ). Additional treatment of asymptomatics (beyond those targeted by current mass screening) is much more likely to lead to control (Figure 2*C* ). In contrast, it may be possible to control VL by treating 90% of symptomatic individuals if asymptomatic and pre-symptomatic infectiousness to sandflies is low. As most *T. cruzi* infections are caused by individuals with asymptomatic Chagas disease, treatment solely of symptomatics is unlikely to lead to a substantial reduction in new infections. The higher reproduction numbers for VL and Chagas mean that it is likely that a higher proportion of pre-symptomatic and/or non-symptomatic infections would need to be treated. Moderate levels of vector control appear sufficient to control gHAT but may not be sufficient on their own to control Chagas disease or VL (Figure 2*D* ). If treatment of symptomatic people is added to vector control it is more likely that transmission of Chagas disease and VL would be interrupted.

**Figure 3.**
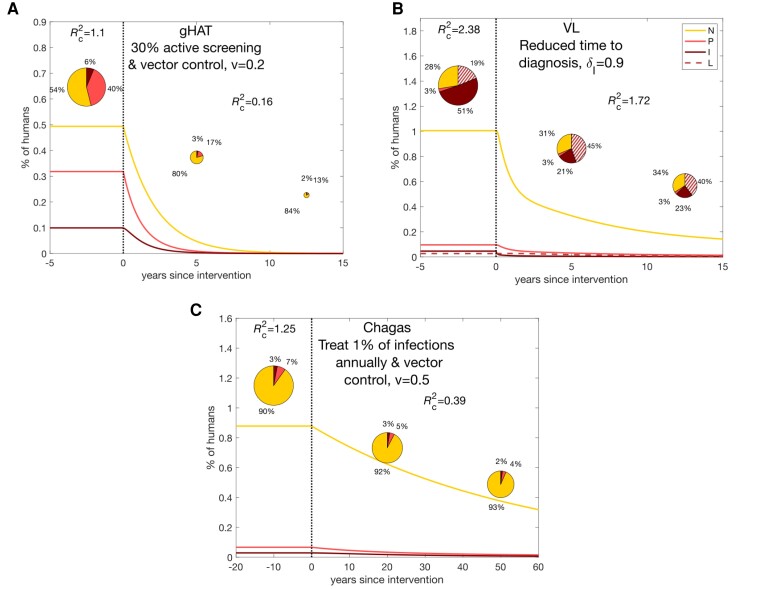
Change in the contribution of non-symptomatic, pre-symptomatic, and symptomatic individuals to transmission over time as interventions are carried out for realistic interventions for each of the 3 diseases. *A*, gHAT. *B*, VL. *C*, Chagas disease. The lines show the proportions of the population in each infection state: non-symptomatic (*N*), pre-symptomatic (*P*), symptomatic (*I*) and, for VL, PKDL (*L*). The pie charts show the contribution of these groups to new infections at particular points on the timeline. *R*_c_^2^ denotes the full-cycle reproduction number accounting for control, 1 − *v* denotes the proportional reduction in the vector-to-host ratio due to vector control (such that 1 indicates no vector control), and *δ*_I_ denotes the proportion of symptomatic infections that are treated before death. Abbreviations: gHAT, *gambiense* human African trypanosomiasis; VL, visceral leishmaniasis.

Simulated interventions against gHAT and Chagas disease reduce transmission substantially, below the critical threshold for elimination, but the reduction in transmission takes a long period of time due to the long timescale of infection (especially for Chagas disease, Figure 3*A* and *C*). Conversely, the strategy for VL is not sufficient for elimination, although transmission reduction happens quite swiftly (Figure 3*B*). The contribution of transmission shifts toward PKDL cases and asymptomatics when interventions target symptomatic VL cases, highlighting the importance of surveillance and treatment of all host groups to achieve elimination (see Supplementary Figures 3–5 for alternative intervention strategies).

## DISCUSSION

Our results suggest that gHAT may be the easiest of the 3 diseases to control with combinations of existing interventions provided there is a functional health system operating in endemic areas. We predict a change from non-symptomatic and pre-symptomatic individuals being responsible for approximately 50% and 40% of overall transmission respectively, to being responsible for roughly 80% and 20% respectively after 5 years. It is important to note that combining interventions targeted at the vector and asymptomatic individuals (active screening), leads to the target being reached more quickly.

For Chagas disease, it is widely known that treatment is more successful for asymptomatics and likely to control their parasite burden. It not only prevents disease progression but also reduces onward transmission in the community leading to a reduction in incidence. Changes in interventions that permit this will only be successful if better diagnostic techniques are developed and more widely used. In particular, diagnosis of asymptomatic hosts and treatment with novel drugs that slow pathogen development and reduce transmission from asymptomatics (rather than attempt to eliminate infection) may prove more viable than only treating overt infections.

The situation for VL is less clear due to fairly limited data on the relative infectiousness of different infection states, the inability to treat asymptomatic cases with currently available drugs, and the challenges of PKDL and HIV coinfection. The literature suggests that VL has a higher reproduction number than Chagas disease and gHAT, and that it is unlikely its transmission can be interrupted without multiple interventions to reduce the time to diagnosis, target the vector, and address the issues of PKDL and asymptomatic reservoirs of infection.

Key to addressing the issue of asymptomatic infection is understanding that infection dynamics are not in equilibrium and that the contribution of different groups to transmission is continually changing. Although it is not possible to capture all aspects of the natural histories and transmission cycles of all vector-borne NTDs in a single model, (eg, possible animal transmission for Chagas and gHAT and maternal transmission for gHAT), the model presented here provides a general framework for considering the role of asymptomatic infection in transmission of vector-borne NTDs that can be built upon for specific diseases, particularly malaria and vector-borne emerging pathogens.

A limitation of all policy-relevant work to date on these diseases is the scarcity of experimental data on which to base assumptions. Our ability to design accurate models could greatly improve with more empirical data using (i) xenodiagnostic techniques where vectors take a bloodmeal from an asymptomatic individual with longitudinal follow-up, (ii) experimental animal infections where vectors take a blood meal from infected animals, and (iii) quantitative comparison with symptomatic patients. A particular challenge that contributes to the uncertainty in the definition of asymptomatic infection is finding good diagnostics for identifying asymptomatics and stratifying them into those who are likely to develop symptoms and those who are not [48–50]. Such diagnostics would enable better patient care and selection of interventions to reduce transmission [38, 51].

This work highlights the currently under-appreciated challenge and importance of asymptomatic infections in three of the world's major NTDs. We have shown that there is potentially a major reservoir of infection in non-symptomatic and pre-symptomatic hosts, and that additional interventions targeted at these groups could lead to considerable progress in the elimination of Chagas, VL, gHAT, and other pathogens. Not only would transmission in the community be reduced, but the burden on the public health system would be lessened if individuals did not progress to disease. Expanding public health programs for these NTDs to focus on systematic testing and treatment of asymptomatic individuals requires a conceptual shift in the underlying control approach, similar to that that occurred during the COVID-19 pandemic, to develop new effective pathways to combat these diseases in which asymptomatic hosts play an important role in transmission.

## Supplementary Data

Supplementary materials are available at *Clinical Infectious Diseases* online. Consisting of data provided by the authors to benefit the reader, the posted materials are not copyedited and are the sole responsibility of the authors, so questions or comments should be addressed to the corresponding author.

## Supplementary Material

ciae096_Supplementary_Data

## Notes

***Author contributions.*** Conceptualization: T. D. H., K. S. R., L. A. C. C., A. P. D., E. R. A., Methodology: K. S. R., L. A. C. C., A. P. D., T. D. H., Software: K. S. R., L. A. C. C., Validation: L. A. C. C. , Formal Analysis: K. S. R., L. A. C. C. Investigation: K. S. R., L. A. C. C., T. D. H, Resources: K. S. R., L. A. C. C., T. D. H., Data Curation: L. A. C. C., K. S. R., Writing—Original Draft Preparation: E. R. A., K. S. R., L. A. C. C., A. P. D., Writing—Reviewing and Editing: K. S. R., L. A. C. C., A. P. D., E. R. A., T. D. H, Visualisation: K. S. R., L. A. C. C., T. D. H, Supervision: E. R. A. , T. D. H, Project administrations: E. R. A., T. D. H, Funding Acquisition: T. D. H.

***Financial support.*** This work was supported by the Bill & Melinda Gates Foundation under the NTD Modelling Consortium project number OPP1053230 (in partnership with the Task Force for Global Health), and project number OPP1184344. K. S. R. also acknowledges funding by the Bill & Melinda Gates Foundation under the HAT MEPP projects OPP1177824 and INV-005121. L. A. C. C. acknowledges funding by the Bill & Melinda Gates Foundation through the Setting the Post-Elimination Agenda for Kala-azar in India consortium (grant number OPP1183986). This supplement is sponsored by funding of Professor T. Déirdre Hollingsworth's research by the Li Ka Shing Foundation at the Big Data Institute, Li Ka Shing Centre for Health Information and Discovery, University of Oxford, and funding of the NTD Modelling Consortium by the Bill & Melinda Gates Foundation (grant number INV-030046). The views, opinions, assumptions, or any other information set out in this article are solely those of the authors and should not be attributed to the funders or any person connected with the funders. The funders had no role in study design, data collection and analysis, decision to publish, or preparation of the manuscript.

***Supplement sponsorship.*** This article appears as part of the supplement “New Tools and Nuanced Interventions to Accelerate Achievement of 2030 Roadmap for Neglected Tropical Diseases,” sponsored by funding of Professor T. Déirdre Hollingsworth's research by the Li Ka Shing Foundation at the Big Data Institute, Li Ka Shing Centre for Health Information and Discovery, University of Oxford; and funding of the NTD Modelling Consortium by the Bill & Melinda Gates Foundation (INV-030046).
